# Gene Expression Profiles of Human Phosphotyrosine Phosphatases Consequent to Th1 Polarisation and Effector Function

**DOI:** 10.1155/2017/8701042

**Published:** 2017-03-14

**Authors:** Patricia Castro-Sánchez, Rocio Ramirez-Munoz, Pedro Roda-Navarro

**Affiliations:** Department of Microbiology I (Immunology), School of Medicine, Complutense University and “12 de Octubre” Health Research Institute, Madrid, Spain

## Abstract

Phosphotyrosine phosphatases (PTPs) constitute a complex family of enzymes that control the balance of intracellular phosphorylation levels to allow cell responses while avoiding the development of diseases. Despite the relevance of CD4 T cell polarisation and effector function in human autoimmune diseases, the expression profile of PTPs during T helper polarisation and restimulation at inflammatory sites has not been assessed. Here, a systematic analysis of the expression profile of PTPs has been carried out during Th1-polarising conditions and upon PKC activation and intracellular raise of Ca^2+^ in effector cells. Changes in gene expression levels suggest a previously nonnoted regulatory role of several PTPs in Th1 polarisation and effector function. A substantial change in the spatial compartmentalisation of ERK during T cell responses is proposed based on changes in the dose of cytoplasmic and nuclear MAPK phosphatases. Our study also suggests a regulatory role of autoimmune-related PTPs in controlling T helper polarisation in humans. We expect that those PTPs that regulate T helper polarisation will constitute potential targets for intervening CD4 T cell immune responses in order to generate new therapies for the treatment of autoimmune diseases.

## 1. Introduction

CD4 T cells are important components of adaptive immune responses. During antigen stimulation, T cells polarise towards a type of effector cell specialised in controlling different sorts of infections by secreting different cytokines: Effector T helper 1 (Th1) secretes IFN*γ* and is specialised against intracellular pathogens, Th2 secretes IL-4 and is specialised against helminths, and Th17 secretes IL-17 and is specialised against extracellular bacterial and fungi. Despite having a crucial role in the immunity against pathogens, helper T cells are also involved in immune system-related diseases, including allergies and autoimmune pathologies. It is well established that Th2 responses mediate allergy and, currently, major efforts are directed to understand the pathological balance of Th1, Th2, and Th17 polarisation in autoimmune diseases [1–4].

In humans, protein tyrosine phosphatases (PTPs) constitute a family of more than 100 enzymes that regulate the phosphorylation state of molecular components of signalling networks. The folding of the PTP domain classifies PTPs in four classes: class I, containing the classical nonreceptor and receptor PTPs (NRPTPs and RPTPs, respectively) and the dual specific phosphatases (DSPs) [5]; class II, containing the low molecular weight PTP (LM-PTP); class III, containing cell division cycle-25 PTPs (CDC25s); and class IV, containing the eyes absent PTPs (EYAs) [6]. Catalytic activity of classes I to III is based on a Cysteine residue, while in the case of class IV it is based on an Aspartic acid residue [5, 6]. Despite their important role in balancing phosphorylation levels, it is becoming clear that they also regulate intracellular signalling by mechanisms not dependent on the phosphatase activity, including the competition for the binding of inhibitors, like in the case of phosphatase of regenerating liver-1 (PRL-1) [7], the control of the spatial regulation of nonphosphorylated substrates, like in the case of MAPK phosphatases (MKPs) [8], and the control of the catalytic activity of other PTPs, like in the case of noncatalytic myotubularins (MTMs) [9]. These mechanisms underscore the relevance of the dose and the spatial regulation of PTPs in the signalling networks that control cell responses.

Lymphocytes express around 60 to 70 genes coding for PTPs [10–12] and the significance of the above-mentioned regulatory mechanisms for the immune responses by human CD4 T cells has been barely established. Studying these mechanisms is needed in order to understand how CD4 T cells achieve normal immune responses while preventing diseases. In this regard, the critical role of some classical PTPs in lymphocyte activation and the association of genetic variants to autoimmune disease have been described [13, 14]. Nonetheless the dose and the regulatory role of the majority of DSPs and class II to IV PTPs (for simplicity called in this study nonclassical PTPs or NCs) in T helper polarisation and effector function have not been studied. Here, we characterise the expression profile of the genes coding for these groups of PTPs in human naïve CD4 T cells, during the polarisation to Th1 effector cells and in response to PKC stimulation and cytosolic raise of Ca^2+^. Our data suggest that changes in the dose of MAPK phosphatases (MKPs) might dramatically affect the regulation of the MAPK module during T cell polarisation and stimulation at the inflammatory sites. Gene expression changes found in our study suggest the existence of previously nonnoted regulators of Th1 polarisation and effector functions and, consequently, potential targets for the manipulation of CD4 T cell immune responses in future research directed to obtain therapies for the treatment of autoimmune diseases.

## 2. Materials and Methods

### 2.1. Cell Isolation, Culture, and Stimulation

Blood cells of healthy adult donors (<65 year old) where obtained from buffy coats processed at the transfusion centre of the “Comunidad de Madrid,” Spain. Peripheral blood mononuclear cells (PBMCs) were obtained by Lymphoprep™ (Rafer, Spain) density gradient centrifugation. Naïve CD4 T cells were isolated from PBMCs using the Naïve CD4^+^ T cell Isolation Kit II (Miltenyi Biotec, Germany). Purities over 95% were typically obtained as assessed by flow cytometry. For Th1 polarising conditions, the obtained naïve CD4 T cells were cultured for 12 days in RPMI 1640 (Lonza Group, Switzerland) supplemented with 10% FCS (Gibco, USA), Penicillin-Streptomycin 100 U/mL and 100 *μ*g/mL, respectively, and 2 mM L-Glutamine (all from Lonza Group, Switzerland) in the presence of Dynabeads Human T-Activator CD3/CD28 (Invitrogen, USA) and 10 ng/ml of IL-12 (Peprotech, USA). Th1 cells were then restimulated for 4 hours with 10 ng/mL Phorbol 12-myristate 13-acetate (PMA) plus 1 *μ*M Ionomycin (Th1-PI) (both from Sigma Aldrich, USA).

### 2.2. Flow Cytometry

Th1 polarisation was assessed by analysing the production of IFN*γ* in response to stimulation with PMA and Ionomycin. Th1 cells were stimulated as explained in Section 2.1 in the presence of 5 *μ*g/mL Brefeldin A (Sigma Aldrich, USA). Cells were then washed, fixed with 4% paraformaldehyde (Sigma Aldrich, USA) permeabilized with 0,1% saponin (Sigma Aldrich, USA), and stained with anti-IFN*γ* antibody (BD pharmingen, USA). Flow cytometry data were collected using a FacsCalibur flow cytometer (BD Biosciences, USA).

### 2.3. Real-Time Quantitative Polymerase Chain Reaction (qPCR)

RNA was extracted from naïve, Th1, and Th1-PI cells using the Absolutely RNA Microprep Kit (Agilent Technologies, USA) and the RNA integrity was assessed using the Agilent 2100 Bioanalyzer (Agilent Technologies, USA). 2 *μ*g of RNA was used to synthesize cDNA with the High Capacity cDNA Reverse Transcription Kit (Applied Biosystems, USA). Expression profiles were obtained by qPCR implemented with TaqMan Low Density Arrays (TLDA, ThermoFisher, USA) in a 7900HT Fast Real-Time PCR System (Applied Biosystems, USA). Genes with CT values under 33 were considered to be nonexpressed. Delta (D)CT was calculated by using as housekeeping gene the average of CT values obtained for the genes 18S and GNB2L1. RQ was calculated using the 2^−ΔΔCT^ method [48].

### 2.4. Data and Statistical Analysis

An agglomerative hierarchical tree was implemented in MATLAB (The Mathworks, Inc, USA) by using Euclidean metrics and the ward method. Statistical analysis was implemented in GraphPad Prism version 5.04 (GraphPad Software, USA). Changes in mRNA levels between different conditions (Naïve, Th1, and Th1-PI) obtained for each donor were analysed with a paired *t*-test. A consistent change in the expression level was considered when it was obtained a difference in expression equal or higher than 1,5 CTs in the majority of donors analysed and a *p* value under 0.1 in the *t*-test. In each data and statistical analysis, only those donors were used in which all the conditions that wanted to be compared were obtained (*n* = 3 donors in analysis of Table 2 and Figures 2 and 3, *n* = 2 in analysis of Figure 4(a) and *n* = 4 donors in the analysis of Figure 4(b)).

## 3. Results and Discussion

### 3.1. Th1 Polarisation of Human Naïve CD4 T Cells

**Figure 1:**
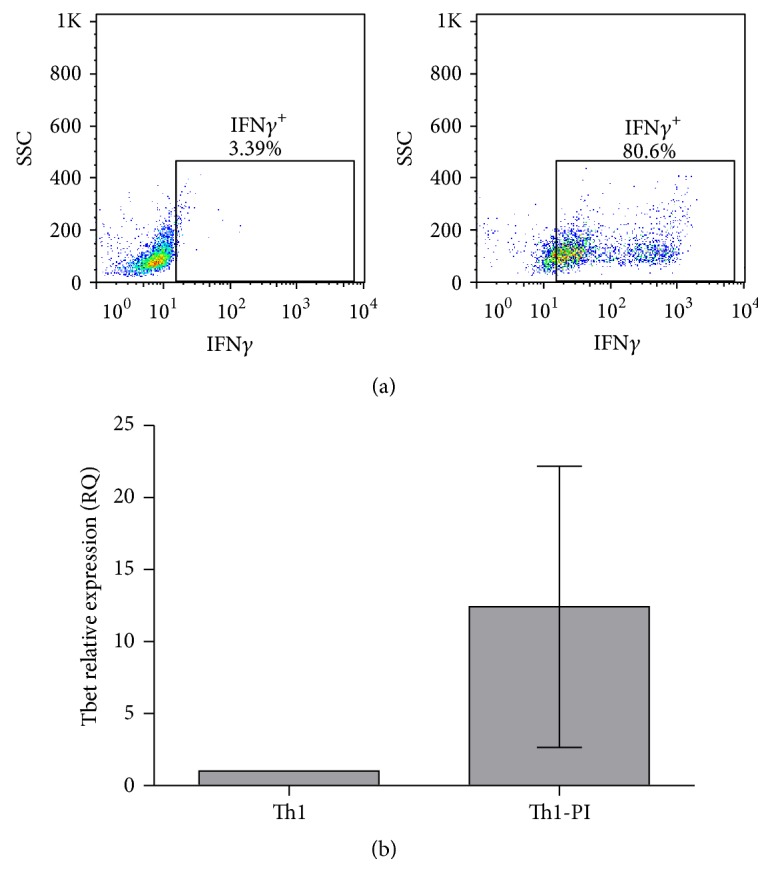
Assessment of Th1 polarisation. (a) Flow cytometry dot plots represent the IFN*γ* production against the size scatter of Th1-polarised cells without (Th1) or with PI treatment (Th1-PI). The percentage of IFN*γ* producing cells is indicated. A representative experiment is shown. (b) Determination of the RQ of the transcription factor Tbet in naïve and Th1 cells. The average and the standard deviations of 3 donors are shown.

Human naïve CD4 T cells were isolated and polarised to Th1 conditions as detailed in materials and methods. The polarisation was confirmed by the production of IFN*γ*. The majority of cells in the population produced IFN*γ* in response to phorbol esters and Ionomycin (PI) treatment (Figure 1(a)). The induction of the Th1 master regulator transcription factor Tbet was also corroborated (Figure 1(b)). PKC activation and cytosolic raise of Ca^2+^, induced by PI treatment, mimic antigen stimulation via the T cell receptor (TCR) during T cell effector functions at inflammatory sites. By using this in vitro model, we studied the amount of mRNA, which indicates the dose of each PTP in naïve and Th1 effector cells.

### 3.2. Expression Profile of PTPs Associated with Human CD4 T Cells

**Figure 2:**
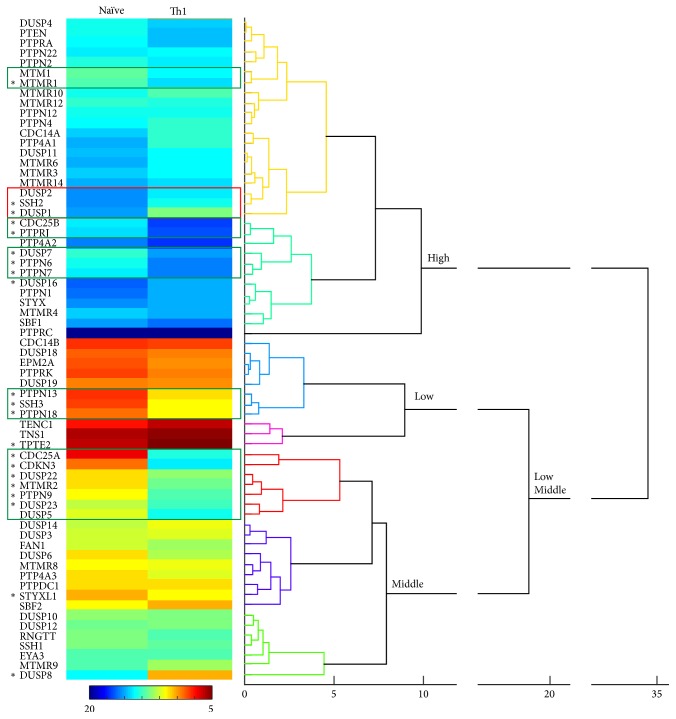
Agglomerative hierarchical tree of the gene expression patterns in naïve and Th1 cells. Numbers below the tree indicate the distance among gene patterns. Hitmap represents the average DCT obtained for each gene in both conditions and 3 donors. The calibration bar is shown between 5 and 20 DCTs. Green and red squares point to clusters of upregulated and downregulated genes, respectively. Asterisks indicate those genes whose expression levels were considered to significantly change, as detailed in Table 2, and explained in materials and methods. Clusters are indicated of high, middle, and low expression.

We included in this study all NCs given the unknown function of the majority of them in T cells and some classical RPTPs and NRPTPs, due to their regulatory role in the signalling downstream the TCR and cytokine receptors and in the dynamics of the cellular machinery or due to their association with autoimmune diseases [13–25, 27, 28, 30–35, 37–39, 49–52] (Table 1). An agglomerative hierarchical tree and statistical analysis were used to characterise the obtained expression profiles (see materials and methods). Consistent with the high number of PTP-coding genes that are expressed in the lymphoid compartment in mice [11], the mRNA of all 14 classical PTPs and 55 out of 65 NCs was detectable in the analysed human naïve and Th1 cells. This fact underscored the relevant role of the family of PTPs in Th1 polarisation and function. Agglomerative hierarchical tree revealed clusters of PTPs, which share not only the expression level and profile during Th1 polarisation but also, in some cases, related functions (Figure 2). The change in the expression profile during Th1 polarisation might be indicative of previously nonnoted regulators of T cell activation/polarisation.

#### 3.2.1. Expression of Classical PTPs

The majority of classical PTPs analysed (10 out of 14) were found inside the group of high expression, including* PTPRC* (CD45),* PTPN1* (PTP1B),* PTPN7* (HePTP),* PTPN6* (SHP1),* PTPRJ* (CD148 or DEP-1),* PTPN4* (MEG1),* PTPN12* (PTP-PEST),* PTPN2* (TC-PTP),* PTPN22* (LYP), and* PTPRA* (PTP-alpha) (Figure 2). Consistent with the established knowledge, PTPRC, an important regulator of Lck activation [14, 16, 17], showed the highest expression levels of all PTPs studied. PTPs found inside the group of middle and low expression included* PTPRK* (PTP-Kappa),* PTPN13* (FAP-1 or PTP-BAS),* PTPN18* (BDP1 or PTP-HSCF), and* PTPN9* (MEG-2) (Figure 2).

*PTPRJ, PTPN6, *and* PTPN7*, which are known to regulate intracellular phosphotyrosine levels during T cell activation [18, 19, 28, 30, 31], were upregulated with Th1 polarisation (Table 2). Consistent with our data, upregulation of* PTPRJ* in response to TCR stimulation has been previously described [18]. Interestingly, mouse Shp1 limits IL-4 signals and then controls abnormal skewing to Th2 polarisation [49]. Therefore, our data indicate that SHP1 might also have a role in balancing Th1/Th2 polarisation in human CD4 T cells.

Our analysis uncovered a relation between the upregulated expression and the function of some PTPs. For example,* PTPN9*, which regulates the fusion of secretory vesicles with the plasma membrane [32, 33], shared cluster with the myotubularin* MTMR2 *(Figure 2), a known regulator of endosomal dynamics (see below). The higher gene expression found in Th1 cells (Figure 2 and Table 2) suggests an important role of these PTPs in endosomal dynamics during the immune responses of Th1 cells. Another example was found inside the group of low expressed PTPs:* PTPN18* and* PTPN13* were found upregulated in the same cluster (Figure 2). Both PTPs have been suggested to regulate* (PTPN18)* or associate with* (PTPN13)* the actin cytoskeleton [50, 51]. Interestingly, they also shared cluster with the DSP Slingshot-3 (SSH3), another regulator of the actin cytoskeleton. Thus, the function of* PTPN18* and* PTPN13* in the actin dynamics subjacent T cell activation should be investigated. Consistent with our data, these PTPs have also been found to be expressed in T cells of mice [11]. Interestingly, a regulatory role of* PTPN13* in Th1 and Th2 polarisation has also been proposed [35].

The genes* PTPN1*,* PTPN2*,* PTPN4*,* PTPN12, PTPN22, PTPRA,* and* PTPRK* were not regulated with Th1-polarising conditions, although the function of some of them in TCR or cytokine signalling has been described (Table 1).

#### 3.2.2. Expression of NCs

We did not detect mRNA of the atypical MKPs* DUSP13*,* DUSP15*,* DUSP26*, and* DUSP27*, the classical MKP* DUSP9*, the tensin homolog* TPTE*, the eyes absent* EYA1*,* EYA2,* and* EYA4,* and the myotubularin* MTMR7*. The reported expression of the* Eya1*,* Eya2,* and* Eya3* mouse orthologs [11] suggests a different requirement of this group of PTPs in mice and humans. The same might apply for the myotubularin* MTMR7*, which seems to have a regulatory role in Th polarisation in mice [53]. The expression of tensin homologs was very low and only* PTEN* was highly expressed particularly in Th1 cells (Figure 2). Although very lowly expressed,* TPTE2* was consistently downmodulated with Th1-polarising conditions (Figure 2 and Table 2). Nonexpressed MKPs in our study matched previous data in mice [11].

**Figure 3:**
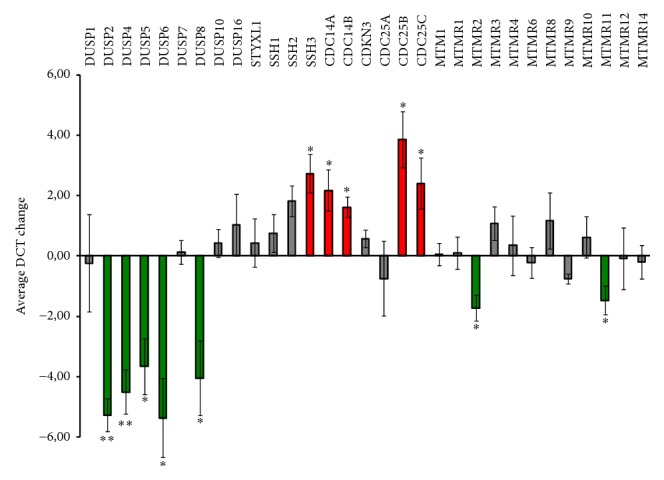
Expression change of NCs phosphatases induced by the PI treatment. The graph represents the average of the change in DCT between Th1 and Th1-PI cells. Genes upregulated and downregulated are labelled in green and red, respectively. Assessment of regulated genes is explained in materials and methods. ^*∗∗*^*p* < 0.01 and ^*∗*^*p* < 0.05 correspond to the probability of paired *t*-test used in comparison of the DCT values obtained in Th1 and Th1-PI cells from 3 donors.

22 NCs were found in the group of highly expressed PTPs (Figure 2). Substantial changes in expression levels associated with Th1 polarisation were found in genes coding for regulators of the phosphorylation state of phosphoinositides (MTMs), the MAPK signalling module (MKPs), the actin cytoskeleton (Slingshots or SSHs), and the cell cycle (CDC25s and CDKN3) (Table 2). Interestingly, the role of some of these enzymes, including MTMR1, MTMR2, DUSP7, DUSP8, DUSP23, STYXL1, and CDC25B, has not been studied in T cells. To investigate the expression profile of these different groups of PTPs during the effector functions of Th1 cells at inflammatory sites, we analysed the change in expression levels induced by the PI treatment (Figure 3).

MTMs dephosphorylate the position 3 of phosphatidylinositol phosphate (PIP) molecules PI(3)P and PI(3,5)P_2_, making them important regulators of the endosomal compartment, the cytoskeleton, and ion channels [9]. Eight out of 12 MTMs were found in the group of highly expressed PTPs (Figure 2), which suggests that they finely tune PIP levels for a proper function of these components of the cellular machinery in T cells. For example, MTMR14 controls the activity of the Ryanodine Receptor (RyR), which is essential for the homeostasis of Ca^2+^ [9]. RyR is needed for Ca^2+^ signalling and proper IL-2 production and proliferation of activated T cells [54]. MTMR14 shared cluster with MTMR6 (Figure 2), which has been described to control naïve CD4 T cell activation by inhibiting the KCa3. 1K^+^ channel, which is essential for Ca^2+^ influx [55]. Consistently, a slightly higher mRNA level was found in naïve T cells than in Th1 cells. Other MTM of this cluster was MTMR3, which, along with MTMR6, has been described to regulate autophagy [9], an essential process for the metabolic changes required during T cell activation [56]. Among the highly expressed MTMs, only MTMR1 was found significantly upregulated (Table 2) and shared cluster with MTM1 (Figure 2), which was also weakly upregulated. MTM1 interacts with desmin [57] and might have an important role in cytoskeleton dynamics in T cells.

We also found phosphatase death (PD) MTMs expressed in CD4 T cells, including the highly expressed SBF1 (MTMR5), MTMR10, and MTMR12, the middle expressed MTMR9, and the inducible with Th1 polarisation MTMR11 (Figure 2 and Table 2). PD MTMs have been found to physically interact and increase the catalytic activity of MTMs [9]. For example, MTMR9 regulates the activity of MTMR6 [9] and MTMR8, a previously described regulator of the PI3K/AKT pathway in zebrafish [58]. MTMR9 might constitute an important regulator of CD4 T cell polarisation as has been proposed in mice [53]. The PI treatment upregulated MTMR11 levels in Th1 cells (Figure 3), suggesting a previously unknown regulatory role in effector functions at inflammatory sites. The MTMs regulated by MTMR11 have not been described.

Inside the group of middle expressed PTPs, MTMR2 was found upregulated by Th1-polarising conditions and PI treatment (Table 2 and Figure 3). These data suggest a previously unknown role of this MTM in T cells during Th1 immune responses. MTMR2 catalytic activity is increased by the PD MTMs SBF1, MTMR12, and SBF2 (MTMR13). Although none of these MTMs were found upregulated upon polarisation, the expression level of SBF1 and MTMR12 seems to be enough to allow this regulatory mechanism to take place in naïve and Th1 cells. MTMR2 dephosphorylates PI(3,5)P, which regulates membrane homeostasis and endosomal transport and has been described to interact with Disc large-1 (Dlg-1), which is involved in polarised membrane trafficking [59]. Recently, it has been proposed that ezrin controls tubulin cytoskeleton dynamics, immunological synapse organization, and NFAT activation by interacting with Dlg-1 [60]. Thus, it is tempting to speculate that MTMR2 has a regulatory role in the dynamics of the endosomal compartment during the activation of Th1 cells at inflammatory sites.

The cytoskeleton regulators SSH2 and SSH3 were found downregulated and upregulated, respectively, by Th1-polarising conditions (Table 2). These data underscore the relevance of SSH3 for the cytoskeleton rearrangements during Th1 polarisation or effector function. Despite both proteins being downmodulated by PI treatment (Figure 3), SSH1 expression was not regulated under any treatment. Interestingly, the role of SSHs, including the middle expressed SSH1, in the intracellular signalling operating during antigen-induced T cell stimulation is not completely understood [61].

Among the 9 out of 10 classical MKPs [8] whose expression was detected, DUSP1, DUSP2, DUSP4, DUSP7, and DUSP16 were found in the group of highly expressed PTPs (Figure 2). Nuclear DUSP1 has been found to be required for proper T cell activation [40] and nuclear/cytoplasmic DUSP16 has been proposed to be downregulated and upregulated during Th1 and Th2 polarisation, respectively, being, in mice, a regulator of the Th1/Th2 balance [41]. Consistently, the expression levels of DUSP16 were found downmodulated during Th1-polarising conditions (Table 2), suggesting a role in balancing human T helper differentiation or in initial T cell immune responses by naïve cells. The cytoplasmic ERK-specific MKP DUSP7 was upregulated with Th1-polarising conditions and shared cluster with the SHP1 and the ERK regulator He-PTP (Table 2 and Figure 2). Interestingly, its function and regulatory mechanism in T cells are not known, and our data might indicate a role of DUSP7 in Th1 polarisation or effector function. The nuclear ERK-specific MKPs DUSP2 and DUSP4 have been described to interact with phosphorylated and dephosphorylated ERK and were upregulated by the PI treatment (Figure 3). Thus, they might be involved in both ERK inactivation and/or accumulation of the dephosphorylated form in the nucleus (see below). DUSP5, DUSP6, DUSP8, and DUSP10 were found inside the group of middle expressed PTPs. Nuclear DUSP5 has been proposed to modulate T cell development and activation [62] and Cytosolic DUSP6 has been proposed to decrease T cell sensitivity by dephosphorylating ERK and, consequently, inhibiting the Lck-ERK positive feedback loop established upon strong T cell stimulation [63]. Interestingly, DUSP5 shared cluster with genes upregulated with polarisation (Figure 2) and both MKPs were upregulated by the PI treatment (Figure 3). The function of the p38- and JNK-specific DUSP8 and DUSP10 in T cell immune responses is not known. DUSP8 was found substantially more abundant in naïve than in Th1 cells (Figure 2) and was upregulated with PI treatment (Figure 3). DUSP10 was not regulated by Th1-polarising conditions or PI treatment.

The group of MKPs are characterised by sharing MAPK substrates. For example, ERK is dephosphorylated by 13 different MKPs. Interestingly, it has been proposed that the spatial distribution of dephosphorylated MAPKs is regulated by the binding of MKPs, such as the nucleocytoplasmic location of ERK by nuclear DUSP5 and cytoplasmic DUSP6, and the accumulation of ERK in the nucleus by DUSP2, DUSP4, and DUSP5 [64–66]. Thus, the coordinated expression levels of MKPs are expected to be essential for the proper function of the MAPK signalling module. Dephosphorylated MAPKs can have functions nondependent on the kinase activity, for example, as transcription factors [8], and they bind with high affinity and increase the activity of MKPs [67]. Thus, more than simply having a role in down-modulating the response of the module, MKPs regulate the subcellular localisation and crosstalk of MAPKs [8, 65]. In this regard, changes in the dose, as may be achieved by regulating their expression levels, may enable MKPs to compete with other molecules for the binding of MAPKs.

**Figure 4:**
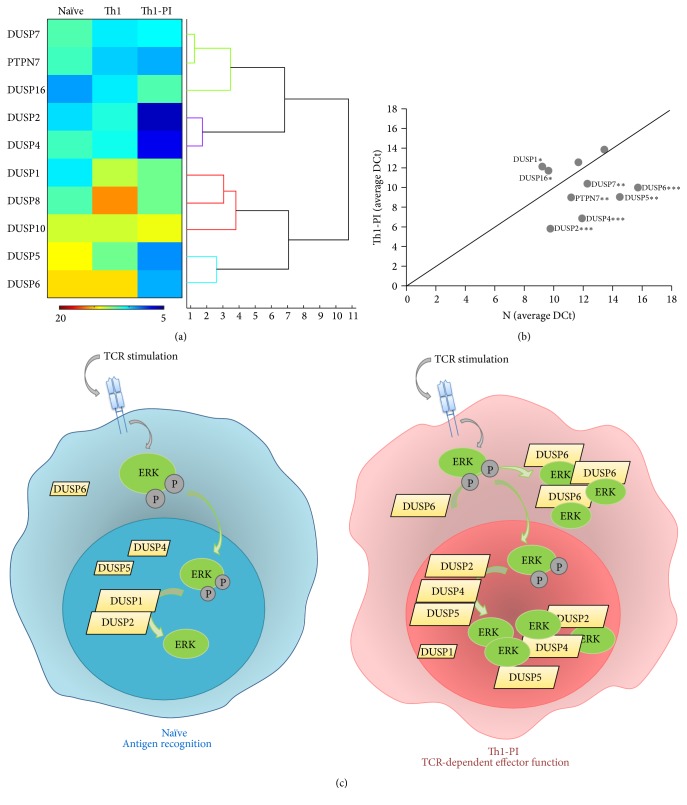
Expression of regulators of the MAPK signalling module in naïve and Th1 cells. (a) Agglomerative hierarchical tree of the expression profile of MAPKs phosphatases in naïve, Th1, and Th1-PI samples. Numbers under the tree indicate the distance among the expression profile. Hitmap represents the average DCT obtained for each gene in all samples and 2 donors. Calibration bar is shown between 5 and 20 DCTs. (b) The average DCT obtained for classical MPKs and PTPN7 in naïve (N) and PI treated Th1 cells (Th1-PI) is plotted. The diagonal line labels the position of genes with equal expression levels in both samples. Labelled genes are those with different expression level assessed as explained in material and methods. ^*∗∗∗*^*p* < 0.001, ^*∗∗*^*p* < 0.01, and ^*∗*^*p* < 0.05 correspond to the probability of paired *t*-test used in comparisons of the DCT values obtained for each gene in naïve and Th1-PI samples from 4 donors. (c) Schematic of proposed spatial regulation of ERK during CD4 T cell immune responses. Dominance of partners of the dephosphorylated ERK in cytoplasm or nucleus might mediate the accumulation of this MAPK and, consequently, promote ERK functions nonmediated by the kinase activity in restimulated Th1 cells.

Consistent with this complex scenario, Th1 polarisation and PI treatment of Th1 cells induced dramatic changes in the expression profile of ERK-directed MKPs and, consequently, remarkable differences were found between naïve and Th1 restimulated cells (Th1-PI) (Figures 4(a) and 4(b)). While the relative expression of nuclear DUSP1 and DUSP2 was dominant in naïve cells, in Th1 restimulated cells there was a clear dominance of partners of dephosphorylated ERK, including DUSP2, DUSP4, and DUSP5 in the nucleus, and DUSP6 in the cytoplasm. These data suggest that interactions between MKPs and dephosphorylated ERK might accumulate ERK in the cytoplasm and/or the nucleus of restimulated Th1 cells, while in naïve cells nuclear translocation and transient phosphorylation of ERK should dominate the response (Figure 4(c)). DUSP7 was also more abundant in restimulated Th1 cells (Figure 4(b)). Whether it can also bind dephosphorylated ERK should be investigated. Thus, functions nonrelated to its kinase activity of dephosphorylated ERK in the cytoplasm and the nucleus of effector T cells at inflammatory sites and the regulatory role of MKPs in the spatial distribution of dephosphorylated ERK should be investigated. Dynamic compartmentalisation of MAPK signalling module by MKPs warrants future research.

Some atypical MKPs were found regulated by Th1-polarising conditions (Table 2). The JNK- and ERK-specific DUSP22 and DUSP23 were found upregulated in our analysis, suggesting an important role in controlling intracellular signalling during Th1 polarisation or effector function. DUSP22 has been proposed to regulate TCR signalling [43] while the function of DUSP23 in T cell activation is not known. The JNK- and p38-specific DUSP13 and DUSP21 (whose substrates are unknown) were repressed with polarisation. The function of these proteins in T cell biology is also unknown.

Among cell cycle regulators, CDC14A and CDC25B were found in the group of highly expressed PTPs. Interestingly, CDC25B, a negative regulator of the cell cycle was upregulated by Th1-polarising conditions (Table 2). CDC25A and CDKN3 constitute a cluster of strongly upregulated cell cycle regulators (Figure 2) and CDC25C was induced with Th1-polarising conditions (Table 2). These data suggest a relevant function of these enzymes for Th1 polarisation, proliferation, or effector function. Consistently, inhibition of T cell proliferation by PD-1 is mediated by suppression of CDC25A [42]. Whether there is a role of these cell cycle regulators during antigen-mediated T cell stimulation, as has been recently described for the CDC25B-specific kinase Aurora A [68], should be investigated.

## 4. Conclusions: Perspective on Autoimmune Diseases

The systematic analysis performed in this study reveals several genes coding for PTPs that are regulated during Th1 polarisation and restimulation of effector cells. Interestingly, the mRNA level of the majority of the regulated genes coding for NCs was increased during Th1 polarisation, which suggests a regulatory role of these PTPs in Th1 polarisation or effector function. By contrast downmodulated genes during polarising conditions might be involved in initial immune responses by naïve T cells. In general, changes in expression levels found during polarisation might indicate a role in achieving a healthy balance of T helper polarisation. Our data also suggest an important compartmentalisation of dephosphorylated ERK functions during the T cell responses at inflammatory sites. Finally, the obtained results also suggest the existence of PTPs that might regulate components of the cellular machinery (including the endosomal compartment, the cytoskeleton, and the activity of ion channels) during T cell immune responses. The regulatory role of these PTPs should be investigated.

Regarding autoimmunity, the expression of* PTPN6* has been found reduced in psoriatic T cells [29] and in blood cells of psoriatic arthritis [69]. Our data suggest that balanced nonpathological T helper polarisation requires a highly upregulated expression of SHP1 during the generation of IFN*γ* producing cells. By contrast, although the polymorphism rs1893217(C) in the* PTPN2* gene has been associated with a decrease in the expression levels that promotes autoimmunity [23, 70] and to a reduced response to IL-2 [26], the regulation of the expression levels of this gene does not seem to be necessary during the generation of IFN*γ* producing cells. Single nucleotide polymorphisms (SNPs) in nonregulated PTPs* PTPN22* and* PTPRC* have been associated with human autoimmune diseases, including multiple sclerosis and autoimmune hepatitis in the case of* PTPRC* and type I diabetes, rheumatoid arthritis, and systemic lupus erythematosus in the case of* PTPN22* [13, 23, 37, 52].

In the mouse model the atypical DUSP22 has been proposed to inhibit TCR signalling and to control the development of experimental autoimmune encephalomyelitis [44]. Interestingly, low expression of DUSP22 in human T cells has been proposed to be a potential biomarker for systemic lupus erythematosus nephritis [45]. Thus, it seems that this protein is relevant in controlling T cell responses in order to prevent autoimmune diseases. Interestingly, overexpression of DUSP23 in CD4 T cells of patients with systemic lupus erythematosus has been described [46]. The upregulation of DUSP22 and DUSP23 found in our work by Th1-polarising conditions suggests a regulatory role of these enzymes during T helper polarisation and, consequently, their contribution to the control or development of pathological polarisation should be investigated. In general, our data encourage comparing the expression profile of PTPs in T cells of patients diagnosed with autoimmune diseases and healthy individuals. Some of the genes regulated during Th1 polarisation as assessed herein might also constitute biomarkers or might be involved in the pathogenesis or severity of autoimmune diseases.

## Figures and Tables

**Table 1:** Class-I classical RPTP and NRPTP included in this study.

Phosphatase	Substrate	Regulation of T cell activation	Involved in autoimmunity	References
*PTPRA* (RPTP*α*)	SFK	Regulation of TCR signalling:	Not reported	[15]
*PTPRC* (CD45)	SFK and JAK family kinases	Regulation of TCR and cytokine signalling	MS, AH	[13, 16, 17]
*PTPRJ* (CD148)	SFK	Regulation of TCR signalling	Not reported	[18, 19]
*PTPRK* (RPTP*κ*)	STAT3	Regulation of CD4 T cell development	Not reported	[20, 21]
*PTPN1* (PTP1B)	STAT6	Not reported	Not reported	[22]
*PTPN2* (TC-PTP)	SFK, JAK1, JAK3	Regulation of TCR and cytokine signalling	T1D, CD, S	[23–26]
*PTPN4* (PTP-MEG1)	CD247	Regulation of TCR signalling	Not reported	[27]
*PTPN6* (SHP1)	SFK, ITAMs, ZAP70, SLP-76	Regulation of TCR signalling	PS	[28, 29]
*PTPN7* (HePTP)	ERK1/2, p38	Regulation of TCR signalling	Not reported	[30, 31]
*PTPN9* (PTP-MEG2)	NSF	Regulation of cytokine secretion	Not reported	[32, 33]
*PTPN12* (PTP-PEST)	Pyk2	Positive regulator of secondary T cell responses	Not reported	[34]
*PTPN13* (PTP-BAS)	STAT4, STAT6	Regulation of cytokine signalling	Not reported	[35]
*PTPN18* (PTP20)	HER2	Not reported	Not reported	[36]
*PTPN22 *(LYP)	ZAP70, LCK, FYN	Regulation of TCR signalling	T1D, RA, SLE	[37–39]

SFK: Src family kinases, MS: multiple sclerosis, AH: autoimmune hepatitis, T1D: type 1 diabetes, CD: Crohn's disease, S: synovitis, PS: psoriasis, RA: rheumatoid arthritis, and SLE: systemic lupus erythematosus.

**Table 2:** PTP regulated during Th1 polarization. PTPs whose expression levels were regulated during Th1 polarization are shown. Changes in expression were determined as explained in materials and methods. Asterisks represent the result of the paired *t*-test of DCt values obtained from the comparison of naïve and Th1 cells in 3 donors. *p* < 0.1 (*∗*), *p* < 0.05 (*∗∗*), or *p* < 0.01 (*∗∗∗*). Absolute values in column 4 indicate the average change in the DCT, generated by subtracting the DCT value obtained for each condition (DCT_Th1 minus DCT_Naïve) in the 3 individual donors analysed, and obtaining the average. DUSP13 and DUSP21 were considered as repressed genes since their expressions were only detectable in naïve but not in Th1 cells. MTMR11 and CDC25C were considered as induced genes since their expression was detectable in Th1 but not in naïve cells.

Group	Phosphatase	Regulation during Th1 polarization	|Average change in DCT|/*p* value	Substrate	Regulation of T cell activation or polarisation/Involvement in autoimmunity
Classical	*PTPRJ* (CD148)	Upregulation	2.02/*∗∗*	SFK	Regulation of TCR signalling/Not reported [18, 19]
	*PTPN6* (SHP1)	Upregulation	2.08/*∗∗*	SFK, ITAMs, ZAP70, SLP-76, Vav1	Regulation of TCR signalling/PS [28, 29]
	*PTPN7* (HePTP)	Upregulation	1.54/*∗∗*	ERK1/2, p38	Regulation of TCR signalling/Not reported [30, 31]
	*PTPN9* (PTP-MEG2)	Upregulation	2.45/*∗∗*	NSF	Regulation of cytokine secretion/Not reported [32, 33]
	*PTPN13* (PTP-BL)	Upregulation	2.40/*∗∗*	STAT4, STAT6	Regulation of cytokine signalling/Not reported [35]
	*PTPN18* (PTP20)	Upregulation	1.9/*∗*	HER2	Not reported/Not reported

MKPs and Atypical DSPs	DUSP1	Downregulation	3.07/*∗∗∗*	p38, JNK, ERK	T cell activation/Not reported [40]
	DUSP7	Upregulation	2.10/*∗*	ERK	Not reported/Not reported
	DUSP8	Downregulation	4.55/*∗∗*	JNK, p38	Not reported/Not reported
	DUSP13	Repression		JNK, p38	Not reported/Not reported
	DUSP16	Downregulation	1.20/*∗*	JNK, p38	Th1/Th2 balance/Not reported [41, 42]
	STYXL1	Upregulation	1.19/*∗*	Catalytically inactive	Not reported/Not reported
	DUSP21	Repression		Unknown	Not reported/Not reported
	DUSP22	Upregulation	2.10/*∗∗*	JNK, ERK2, Lck	Regulation of TCR signaling/EAE, SLE [43–45]
	DUSP23	Upregulation	1.57/*∗∗∗*	p38, JNK	Not reported/SLE [46]

Myotubularins	MTMR1	Upregulation	1.43/*∗∗∗*	PI(3)P, PI(3,5)P2	Not reported/Not reported
	MTMR2	Upregulation	2.61/*∗∗*	PI(3)P, PI(3,5)P2	Not reported/Not reported
	MTMR11	Induction		Catalytically inactive	Not reported/Not reported

SSHs, CDC14s and PTEN DSPs	SSH2	Downregulation	1.68/*∗∗*	Cofilin	Not reported/Not reported
	SSH3	Upregulation	2.63/*∗∗∗*	Cofilin	Not reported/Not reported
	TPTE2	Downregulation	1.96/*∗*	PIP	Not reported/Not reported
	CDKN3	Upregulation	5.81/*∗∗*	CDK2	Inhibition of cell cycle/Not reported [47]

Class III Cys-based PTPs	CDC25A	Upregulation	6.80/*∗∗*	CDKs	Promotion of cell cycle/Not reported [42]
	CDC25B	Upregulation	2.45/*∗∗*	CDKs	Not reported/Not reported
	CDC25C	Induction		CDKs	Not reported/Not reported

PS: psoriasis. EAE: experimental autoimmune encephalomyelitis. SLE: systemic lupus erythematosus.

## References

[1] Fournier C. (2005). Where do T cells stand in rheumatoid arthritis?. *Joint Bone Spine*.

[2] Baccala R., Kono D. H., Theofilopoulos A. N. (2005). Interferons as pathogenic effectors in autoimmunity. *Immunological Reviews*.

[3] Miyake K., Akahoshi M., Nakashima H. (2011). Th subset balance in lupus nephritis. *Journal of Biomedicine and Biotechnology*.

[4] Diani M., Altomare G., Reali E. (2016). T helper cell subsets in clinical manifestations of psoriasis. *Journal of Immunology Research*.

[5] Tonks N. K. (2006). Protein tyrosine phosphatases: from genes, to function, to disease. *Nature Reviews Molecular Cell Biology*.

[6] Alonso A., Sasin J., Bottini N. (2004). Protein tyrosine phosphatases in the human genome. *Cell*.

[7] Bai Y., Luo Y., Liu S. (2011). PRL-1 protein promotes ERK1/2 and RhoA protein activation through a non-canonical interaction with the Src homology 3 domain of p115 Rho GTpase-activating protein. *The Journal of Biological Chemistry*.

[8] Caunt C. J., Keyse S. M. (2013). Dual-specificity MAP kinase phosphatases (MKPs): shaping the outcome of MAP kinase signalling. *FEBS Journal*.

[9] Hnia K., Vaccari I., Bolino A., Laporte J. (2012). Myotubularin phosphoinositide phosphatases: cellular functions and disease pathophysiology. *Trends in Molecular Medicine*.

[10] Mustelin T., Vang T., Bottini N. (2005). Protein tyrosine phosphatases and the immune response. *Nature Reviews Immunology*.

[11] Arimura Y., Yagi J. (2010). Comprehensive expression profiles of genes for protein tyrosine phosphatases in immune cells. *Science Signaling*.

[12] Hijikata A., Kitamura H., Kimura Y. (2007). Construction of an open-access database that integrates cross-reference information from the transcriptome and proteome of immune cells. *Bioinformatics*.

[13] Vang T., Miletic A. V., Arimura Y., Tautz L., Rickert R. C., Mustelin T. (2008). Protein tyrosine phosphatases in autoimmunity. *Annual Review of Immunology*.

[14] Rhee I., Veillette A. (2012). Protein tyrosine phosphatases in lymphocyte activation and autoimmunity. *Nature Immunology*.

[15] Maksumova L., Wang Y., Wong N. K. Y., Le H. T., Pallen C. J., Johnson P. (2007). Differential function of PTP*α* and PTP*α* Y789F in T cells and regulation of PTP*α* phosphorylation at Tyr-789 by CD45. *The Journal of Biological Chemistry*.

[16] Mustelin T., Coggeshall K. M., Altman A. (1989). Rapid activation of the T-cell tyrosine protein kinase pp56lck by the CD45 phosphotyrosine phosphatase. *Proceedings of the National Academy of Sciences of the United States of America*.

[17] Irie-Sasaki J., Sasaki T., Matsumoto W. (2001). CD45 is a JAK phosphatase and negatively regulates cytokine receptor signalling. *Nature*.

[18] Lin J., Weiss A. (2003). The tyrosine phosphatase CD148 is excluded from the immunologic synapse and down-regulates prolonged T cell signaling. *Journal of Cell Biology*.

[19] Baker J. E., Majeti R., Tangye S. G., Weiss A. (2001). Protein tyrosine phosphatase CD148-mediated inhibition of T-cell receptor signal transduction is associated with reduced LAT and phospholipase C*γ*1 phosphorylation. *Molecular and Cellular Biology*.

[20] Erdenebayar N., Maekawa Y., Nishida J., Kitamura A., Yasutomo K. (2009). Protein-tyrosine phosphatase-kappa regulates CD4+ T cell development through ERK1/2-mediated signaling. *Biochemical and Biophysical Research Communications*.

[21] Iwata R., Sasaki N., Agui T. (2010). Contiguous gene deletion of Ptprk and Themis causes T-helper immunodeficiency (thid) in the LEC rat. *Biomedical Research*.

[22] Lu X., Malumbres R., Shields B. (2008). PTP1B is a negative regulator of interleukin 4-induced STAT6 signaling. *Blood*.

[23] Doody K. M., Bussières-Marmen S., Li A., Paquet M., Henderson J. E., Tremblay M. L. (2012). T cell protein tyrosine phosphatase deficiency results in spontaneous synovitis and subchondral bone resorption in mice. *Arthritis and Rheumatism*.

[24] Wiede F., Shields B. J., Chew S. H. (2011). T cell protein tyrosine phosphatase attenuates T cell signaling to maintain tolerance in mice. *Journal of Clinical Investigation*.

[25] Simoncic P. D., Lee-Loy A., Barber D. L., Tremblay M. L., McGlade C. J. (2002). The T cell protein tyrosine phosphatase is a negative regulator of Janus family kinases 1 and 3. *Current Biology*.

[26] Long S. A., Cerosaletti K., Wan J. Y. (2011). An autoimmune-associated variant in PTPN2 reveals an impairment of IL-2R signaling in CD4+ T cells. *Genes and Immunity*.

[27] Young J. A., Becker A. M., Medeiros J. J. (2008). The protein tyrosine phosphatase PTPN4/PTP-MEG1, an enzyme capable of dephosphorylating the TCR ITAMs and regulating NF-*κ*B, is dispensable for T cell development and/or T cell effector functions. *Molecular Immunology*.

[28] Brockdorff J., Williams S., Couture C., Mustelin T. (1999). Dephosphorylation of ZAP-70 and inhibition of T cell activation by activated SHP1. *European Journal of Immunology*.

[29] Eriksen K. W., Woetmann A., Skov L. (2010). Deficient SOCS3 and SHP-1 expression in psoriatic T cells. *Journal of Investigative Dermatology*.

[30] Nika K., Charvet C., Williams S. (2006). Lipid raft targeting of hematopoietic protein tyrosine phosphatase by protein kinase C *θ*-mediated phosphorylation. *Molecular and Cellular Biology*.

[31] Saxena M., Williams S., Brockdorff J., Gilman J., Mustelin T. (1999). Inhibition of T cell signaling by mitogen-activated protein kinase-targeted hematopoietic tyrosine phosphatase (HePTP). *Journal of Biological Chemistry*.

[32] Huynh H., Bottini N., Williams S. (2004). Control of vesicle fusion by a tyrosine phosphatase. *Nature Cell Biology*.

[33] Wang Y., Vachon E., Zhang J. (2005). Tyrosine phosphatase MEG2 modulates murine development and platelet and lymphocyte activation through secretory vesicle function. *Journal of Experimental Medicine*.

[34] Davidson D., Shi X., Zhong M.-C., Rhee I., Veillette A. (2010). The phosphatase PTP-PEST promotes secondary t cell responses by dephosphorylating the protein tyrosine kinase Pyk2. *Immunity*.

[35] Nakahira M., Tanaka T., Robson B. E., Mizgerd J. P., Grusby M. J. (2007). Regulation of signal transducer and activator of transcription signaling by the tyrosine phosphatase PTP-BL. *Immunity*.

[36] Wang H.-M., Xu Y.-F., Ning S.-L. (2014). The catalytic region and PEST domain of PTPN18 distinctly regulate the HER2 phosphorylation and ubiquitination barcodes. *Cell research*.

[37] Stanford S. M., Mustelin T. M., Bottini N. (2010). Lymphoid tyrosine phosphatase and autoimmunity: human genetics rediscovers tyrosine phosphatases. *Seminars in Immunopathology*.

[38] Wu J. S., Katrekar A., Honigberg L. A. (2006). Identification of substrates of human protein-tyrosine phosphatase PTPN22. *The Journal of Biological Chemistry*.

[39] Stanford S. M., Bottini N. (2014). PTPN22: the archetypal non-HLA autoimmunity gene. *Nature Reviews Rheumatology*.

[40] Zhang Y., Reynolds J. M., Chang S. H. (2009). MKP-1 is necessary for T cell activation and function. *Journal of Biological Chemistry*.

[41] Musikacharoen T., Bandow K., Kakimoto K. (2011). Functional involvement of Dual Specificity Phosphatase 16 (DUSP16), a c-Jun N-terminal kinase-specific phosphatase, in the regulation of T helper cell differentiation. *Journal of Biological Chemistry*.

[42] Patsoukis N., Sari D., Boussiotis V. A. (2012). PD-1 inhibits T cell proliferation by upregulating p27 and p15 and suppressing Cdc25A. *Cell Cycle*.

[43] Alonso A., Merlo J. J., Na S. (2002). Inhibition of T cell antigen receptor signaling by VHR-related MKPX (VHX), a new dual specificity phosphatase related to VH1 related (VHR). *Journal of Biological Chemistry*.

[44] Li J.-P., Yang C.-Y., Chuang H.-C. (2014). The phosphatase JKAP/DUSP22 inhibits T-cell receptor signalling and autoimmunity by inactivating Lck. *Nature Communications*.

[45] Chuang H. C., Chen Y. M., Hung W. T. (2016). Downregulation of the phosphatase JKAP/DUSP22 in T cells as a potential new biomarker of systemic lupus erythematosus nephritis. *Oncotarget*.

[46] Balada E., Felip L., Ordi-Ros J., Vilardell-Tarres M. (2017). DUSP23 is over-expressed and linked to the expression of DNMTs in CD4^+^ T cells from systemic lupus erythematosus patients. *Clinical & Experimental Immunology*.

[47] Chen C.-F., Feng X., Liao H.-Y. (2014). Regulation of T cell proliferation by JMJD6 and PDGF-BB during chronic hepatitis B infection. *Scientific Reports*.

[48] Livak K. J., Schmittgen T. D. (2001). Analysis of relative gene expression data using real-time quantitative PCR and the 2-ΔΔCT method. *Methods*.

[49] Johnson D. J., Pao L. I., Dhanji S., Murakami K., Ohashi P. S., Neel B. G. (2013). Shp1 regulates T cell homeostasis by limiting IL-4 signals. *Journal of Experimental Medicine*.

[50] Shiota M., Tanihiro T., Nakagawa Y. (2003). Protein tyrosine phosphatase PTP20 induces actin cytoskeleton reorganization by dephosphorylating p190 RhoGAP in rat ovarian granulosa cells stimulated with follicle-stimulating hormone. *Molecular Endocrinology*.

[51] Van Ham M., Kemperman L., Wijers M., Fransen J., Hendriks W. (2005). Subcellular localization and differentiation-induced redistribution of the protein tyrosine phosphatase PTP-BL in neuroblastoma cells. *Cellular and Molecular Neurobiology*.

[52] Wellcome Trust Case Control Consortium (2007). Genome-wide association study of 14,000 cases of seven common diseases and 3,000 shared controls. *Nature*.

[53] Guo L., Martens C., Bruno D. (2013). Lipid phosphatases identified by screening a mouse phosphatase shRNA library regulate T-cell differentiation and Protein kinase B AKT signaling. *Proceedings of the National Academy of Sciences of the United States of America*.

[54] Dadsetan S., Zakharova L., Molinski T. F., Fomina A. F. (2008). Store-operated Ca^2+^ influx causes Ca^2+^ release from the intracellular Ca^2+^ channels that is required for T cell activation. *Journal of Biological Chemistry*.

[55] Srivastava S., Ko K., Choudhury P. (2006). Phosphatidylinositol-3 phosphatase myotubularin-related protein 6 negatively regulates CD4 T cells. *Molecular and Cellular Biology*.

[56] Bronietzki A. W., Schuster M., Schmitz I. (2015). Autophagy in T-cell development, activation and differentiation. *Immunology and Cell Biology*.

[57] Hnia K., Tronchère H., Tomczak K. K. (2011). Myotubularin controls desmin intermediate filament architecture and mitochondrial dynamics in human and mouse skeletal muscle. *The Journal of Clinical Investigation*.

[58] Mei J., Li Z., Gui J.-F. (2009). Cooperation of Mtmr8 with PI3K regulates actin filament modeling and muscle development in zebrafish. *PLoS ONE*.

[59] Bolino A., Bolis A., Previtali S. C. (2004). Disruption of *Mtmr2* produces CMT4B1-like neuropathy with myelin outfolding and impaired spermatogenesis. *Journal of Cell Biology*.

[60] Lasserre R., Charrin S., Cuche C. (2010). Ezrin tunes T-cell activation by controlling Dlg1 and microtubule positioning at the immunological synapse. *The EMBO Journal*.

[61] Ramirez-Munoz R., Castro-Sánchez P., Roda-Navarro P. (2016). Ultrasensitivity in the cofilin signaling module: a mechanism for tuning T cell responses. *Frontiers in Immunology*.

[62] Moon S.-J., Lim M.-A., Park J.-S. (2014). Dual-specificity phosphatase 5 attenuates autoimmune arthritis in mice via reciprocal regulation of the Th17/Treg cell balance and inhibition of osteoclastogenesis. *Arthritis & Rheumatology*.

[63] Acuto O., Bartolo V. D., Michel F. (2008). Tailoring T-cell receptor signals by proximal negative feedback mechanisms. *Nature Reviews Immunology*.

[64] Rodriguez J., Crespo P. (2011). Working without kinase activity: phosphotransfer-independent functions of extracellular signal-regulated kinases. *Science Signaling*.

[65] Caunt C. J., Armstrong S. P., Rivers C. A., Norman M. R., McArdle C. A. (2008). Spatiotemporal regulation of ERK2 by dual specificity phosphatases. *Journal of Biological Chemistry*.

[66] Mandl M., Slack D. N., Keyse S. M. (2005). Specific inactivation and nuclear anchoring of extracellular signal-regulated kinase 2 by the inducible dual-specificity protein phosphatase DUSP5. *Molecular and Cellular Biology*.

[67] Zhou B., Zhang Z.-Y. (1999). Mechanism of mitogen-activated protein kinase phosphatase-3 activation by ERK2. *Journal of Biological Chemistry*.

[68] Blas-Rus N., Bustos-Morán E., de Castro I. P. (2016). Aurora A drives early signalling and vesicle dynamics during T-cell activation. *Nature Communications*.

[69] Batliwalla F. M., Li W., Ritchlin C. T. (2005). Microarray analyses of peripheral blood cells identifies unique gene expression signature in psoriatic arthritis. *Molecular Medicine*.

[70] Wiede F., Sacirbegovic F., Leong Y. A., Yu D., Tiganis T. (2017). PTPN2-deficiency exacerbates T follicular helper cell and B cell responses and promotes the development of autoimmunity. *Journal of Autoimmunity*.

