# Corrigendum to “Gene Expression Profiles of Human Phosphotyrosine Phosphatases Consequent to Th1 Polarisation and Effector Function”

**DOI:** 10.1155/2017/9031753

**Published:** 2017-12-10

**Authors:** Patricia Castro-Sánchez, Rocio Ramirez-Munoz, Pedro Roda-Navarro

**Affiliations:** Department of Microbiology I (Immunology), School of Medicine, Complutense University and “12 de Octubre” Health Research Institute, Madrid, Spain

In the article titled “Gene Expression Profiles of Human Phosphotyrosine Phosphatases Consequent to Th1 Polarisation and Effector Function” [1], there was an error in Figure 2 where the calibration bar should indicate 20 in the red side and 5 in the blue side. The correct figure is as follows.

## Figures and Tables

**Figure 2:**
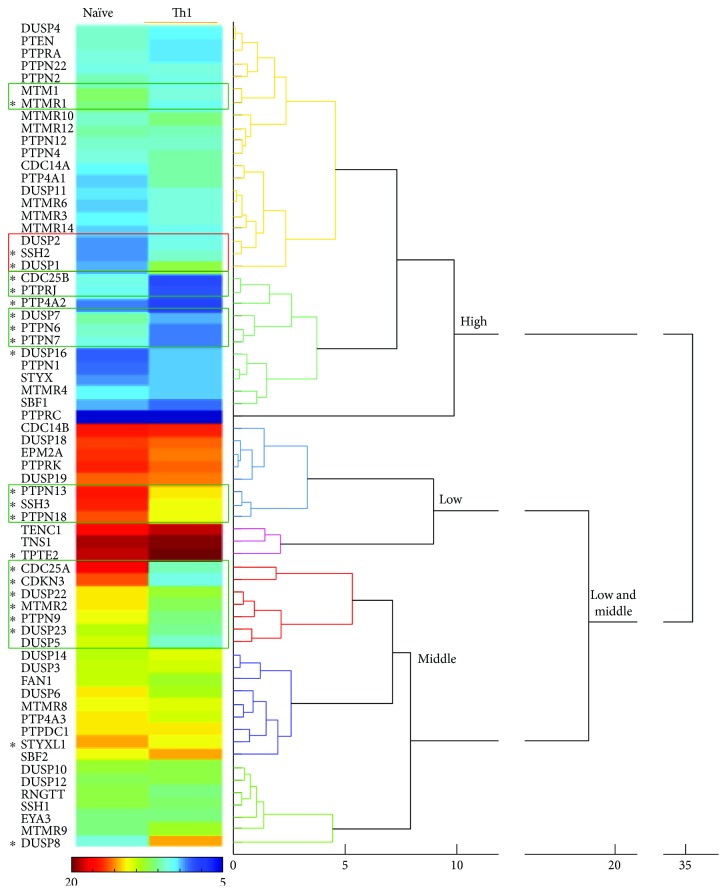
Agglomerative hierarchical tree of the gene expression patterns in naïve and Th1 cells. Numbers below the tree indicate the distance among gene patterns. Heat map represents the average DCT obtained for each gene in both conditions and 3 donors. The calibration bar is shown between 5 and 20 DCTs. Green and red squares point to clusters of upregulated and downregulated genes, respectively. Asterisks indicate those genes whose expression levels were considered to significantly change, as detailed in Table 2, and explained in Materials and Methods. Clusters are indicated of high, middle, and low expression.
